# Optimising the Delivery of RHDV to Rabbits for Biocontrol: An Experimental Evaluation of Two Novel Methods of Virus Delivery

**DOI:** 10.3390/v15091814

**Published:** 2023-08-25

**Authors:** Tshewang Dorji, Ridma M. J. Jayasingha Ellakkala Appuhamilage, Peter L. Bird, Nina Huang, Tiffany W. O’Connor, Kandarp K. Patel, Tanja Strive, Patrick L. Taggart

**Affiliations:** 1 Biosecurity, Department of Primary Industries and Regions (PIRSA), Urrbrae, SA 5064, Australia; tshewang.dorji@adelaide.edu.au (T.D.); ridmajayasingha@yahoo.com (R.M.J.J.E.A.); kandarp.patel@adelaide.edu.au (K.K.P.); 2 School of Food, Agriculture and Wine, The University of Adelaide, Urrbrae, SA 5064, Australia; 3 Waite Conservation Reserve, University of Adelaide, Urrbrae, SA 5064, Australia; pbjbird1@bigpond.com; 4 Health & Biosecurity, Commonwealth Scientific and Industrial Research Organisation, Acton, ACT 2601, Australia; nina.huang@csiro.au (N.H.); tanja.strive@csiro.au (T.S.); 5 Virology Laboratory, Elizabeth Macarthur Agricultural Institute, Department of Primary Industries NSW, Menangle, NSW 2568, Australia; 6 School of Animal and Veterinary Sciences, The University of Adelaide, Roseworthy, SA 5371, Australia; 7 Vertebrate Pest Research Unit, Department of Primary Industries NSW, Queanbeyan, NSW 2620, Australia; 8 School of Biological, Earth and Environmental Sciences, University of New South Wales, Sydney, NSW 2000, Australia

**Keywords:** rabbit haemorrhagic disease virus, survival, *Oryctolagus cuniculus*, biological control, bait

## Abstract

Rabbit haemorrhagic disease virus (RHDV) is established as a landscape-scale biocontrol that assists the management of invasive European rabbits and their impacts in both Australia and New Zealand. In addition to this, it is also available to land managers to augment rabbit control efforts at a local scale. However, current methods of deploying RHDV to rabbits that rely on the consumption of virus-treated baits can be problematic as rabbits are reluctant to consume bait when there is abundant, green, protein-rich feed available. We ran a suite of interrupted time-series experiments to compare the duration of infectivity of two conventional (carrot and oat baits) and two novel (meat bait and soil burrow spray) methods of deploying RHDV to rabbits. All methods effectively killed exposed rabbits. Soil burrow spray and carrot baits resulted in infection and mortality out to 5 days post their deployment in the field, and meat baits caused infection out to 10 days post their deployment. In contrast, oat baits continued to infect and kill exposed rabbits out to 20 days post deployment. Molecular assays demonstrated high viral loads in deployed baits beyond the duration for which they were infectious or lethal to rabbits. Based on our results, we suggest that the drying of meat baits may create a barrier to effective transmission of RHDV by adult flies within 10 days. We therefore hypothesise that fly larvae production and development on infected tissues is critical to prolonged viral transmission from meat baits, and similarly from carcasses of RHDV mortalities, via mechanical fly vectors. Our study demonstrates that meat baits and soil spray could provide additional virus deployment options that remove the need for rabbits to consume baits at times when they are reluctant to do so.

## 1. Introduction

The European rabbit (*Oryctolagus cuniculus*) is an environmentally and economically significant pest animal in Australia. The environmental impact of invasive rabbit populations are wide and varied, including direct herbivory and an associated decrease in the regeneration of native vegetation, competition with native fauna for food resources, land degradation through increased erosion and weed infestation, and supporting large populations of introduced predators [1,2]. These wide and varied impacts contribute to their large economic costs for the agricultural sectors [3,4,5].

Two biocontrol agents, myxoma virus and rabbit haemorrhagic disease virus (RHDV), have proven to be effective means of managing invasive rabbit populations at landscape scales [6,7,8]. Both lead to fatal disease and cause myxomatosis and rabbit haemorrhagic disease (RHD), respectively. The success of both viruses is mostly due to their ability to self-disseminate and act continuously at continental scales [8,9,10]. However, while myxoma virus and RHDV have had a substantial, long-term impact on invasive rabbits, they do not achieve complete control, and thus, land managers must employ additional methods of population management.

At local scales, tools available to land managers to control rabbit populations include a combination of conventional control and biocontrol methods. Conventional control methods include warren ripping, aboveground harbour destruction, poison baiting, warren fumigation, shooting, and trapping. In contrast, commercial access to RHDV for its release by land managers is the only biocontrol method available for local application, as myxoma virus is no longer accessible or released. The idea of local releases of virus, in addition to endemic RHDV circulation at continental scales, is to initiate disease outbreaks earlier or more frequently than would otherwise happen because of outbreaks of naturally circulating RHDV.

Since 2016, local releases of RHDV for the control of residual rabbit populations have increased substantially, with suggestions that land managers view them as a ‘silver bullet’ control—the only control required to successfully manage populations [11] (Taggart et al. 2023 IN PRESS J Pest Science). RHDV release is cheaper and both time and labour efficient relative to most conventional control methods; it is also safe for nontarget species if accidentally consumed [12]. However, virus release is not without challenges. Current legislation in Australia stipulates that RHDV must be released either by inoculation and release of a wild rabbit or via carrot or oat baits [13]. This requires either (1) a wild rabbit to be successfully trapped, inoculated with the virus, and then released back into the warren or (2) the consumption of the carrot or oat baits by rabbits around target warrens.

These methods of delivering RHDV to rabbits have several obvious disadvantages. The first method requires the appropriate equipment to capture wild rabbits alive and unharmed. Trapping can also be labour and time intensive when population densities are low and successful trapping relies on animals being actively interested in and attracted to the trap bait. Furthermore, trapping is known to be biased towards younger animals, which can have a higher chance of surviving RHDV infections [14]. This would undoubtedly be problematic if releasing RHDV via inoculation into wild rabbits. Similarly, the delivery of RHDV on carrot or oat baits also relies on rabbits consuming virus-treated bait. This is problematic as rabbits are reluctant to consume bait when there is abundant, green, protein-rich feed available [15].

To compound the problems with bait delivery, the release of RHDV by land managers often does not follow strict product labels or recommendations [11] (Taggart et al. 2023 IN PRESS J Pest Science). Although the RHDV product label stipulates that carrot or oat baits are distributed at dusk and remaining bait is recovered and destroyed the following morning, anecdotal reports suggest that baits commonly remain in situ for prolonged periods. This is problematic as RHDV is known to be inactivated by UV radiation, and hence, transmission from ground-laid baits in direct sunlight may be limited [16]. There is therefore a need to develop novel methods of delivering RHDV to rabbits that do not solely rely on active bait consumption and seek to maximise both the likelihood of infection and the period over which transmission of RHDV to rabbits can occur.

Under natural conditions, broad-scale transmission of RHDV is largely thought to occur through contaminated mechanical vectors, such as flies [17,18,19,20]. However, once RHDV is introduced into a rabbit population by flies, the dominant transmission pathway within the population is thought to be by rabbits becoming contaminated with the virus themselves, which is then ingested while grooming. This would be expected as the faecal route is known to be the main source of disseminating RHDV, increasing the probability that rabbits themselves become contaminated with the virus when visiting communal latrines [21,22]. Similarly, RHDV is excreted in nasal secretions and would be expected to contaminate warren soils [21]. Rabbits also frequently die of RHDV inside warrens, creating virus-laden carcasses within the warren system that are frequented by flies and further contribute to the contamination of the warren itself [9]. Rabbits in contact with contaminated latrines and warren soils would become infected through natural grooming behaviours.

With natural RHDV transmission pathways in mind, we assessed two novel methods of delivering RHDV to rabbits: (1) a meat bait that simulated an RHDV-laden rabbit carcass to deliver RHDV to rabbits via fly vectors; and (2) a soil burrow spray, used to contaminate burrow entrances and deliver RHDV to rabbits via their natural grooming behaviours. We compared the duration of infectivity of these two novel methods (meat bait and soil spray) to conventional delivery methods (carrot and oat bait) in susceptible laboratory rabbits (*Oryctolagus cuniculus*). We predict that our novel methods of RHDV release and delivery to rabbits will have implications for the use of RHDV as a local biocide, particularly where delivery to rabbits may be challenging.

## 2. Materials and Methods

### 2.1. Experiment Design

We tested the infectivity of five different RHDV treatments to rabbits: (1) meat bait; (2) soil spray; (3) carrot bait; (4) oat bait; and (5) negative control bait. The infectivity of each treatment was assessed at nine different time points: 1, 5, 10, 20, 40, 60, 80, 100, and 120 days post deployment. We simultaneously deployed all treatments at the same site at the Waite Conservation Reserve, South Australia, and all were therefore subject to equivalent environmental conditions. We fenced our field site to prevent access to baits by animals. Over the experimental period (30 August 2022–14 November 2022), the average maximum daily temperature was 14.0 °C (range: 7.7, 26.1; Mount Lofty weather station, Bureau of Meteorology) with an average daily rainfall of 2.8 mm (total rainfall 214 mm; Brownhill Creek (Scotch College) weather station, Bureau of Meteorology).

We then tested the infectivity of the deployment method at each timepoint by exposure to susceptible laboratory rabbits. We used nine rabbits across the five deployment methods at each timepoint, with two for each of the four RHDV treatments and one for a negative control. Rabbits were exposed via an equivalent route through which transmission would occur if a particular method was deployed to wild rabbit populations. That is, the meat bait relied on fly transmission, soil spray relied on transmission via natural grooming behaviours, and both the carrot and oat baits relied on oral exposure. Mortality and disease consistent with RHDV in inoculated rabbits, or seroconversion and evidence of RHDV in the rabbit’s liver, were used to demonstrate that a given deployment method remained infectious at a given time point. Testing of each deployment method was terminated after no rabbit deaths were observed for two consecutive time-point trials.

### 2.2. Laboratory Rabbits

We sourced sub-adult New Zealand white rabbits (8–10 weeks of age) from a research colony at Flinders University, South Australia. All rabbits were transported to a laboratory at PIRSA Biosecurity, Urrbrae, South Australia, and individually housed in enclosed boxes with insect-proof mesh lids. We provided rabbits with water and food (commercial rabbit pellets and fresh carrots) ad libitum and allowed them to acclimatise to their new environment for at least two weeks prior to their inclusion in exposure trials. This made all rabbits >10 weeks of age at the time of the first inoculation trials. We changed rabbit bedding prior to bait exposure but not thereafter to limit the possibility of virus transfer between rabbits. Our facility containing the individually housed rabbits was temperature controlled (10–25 °C) with natural lighting, but was not an approved biosafety laboratory.

### 2.3. Deployment Methods

The meat baits, in open plastic containers, and soil spray were each deployed into 18 simulated rabbit burrows (36 burrows total) dug into the side of a hill at our field site. Simulated burrows were 20 cm in diameter and 80 cm deep. This was predicted to reduce direct exposure of the virus to UV radiation and consequently increase its longevity [17]. Deployment of meat baits inside experimental burrows was also necessary to conform with legislation restricting consumption of animal material by ruminants, and for the soil spray, it was necessary to conform with the intended mode of RHDV transmission from soil, described below. In contrast, carrot and oat baits were both deployed on the ground in open plastic containers in direct sunlight, similar to how such baits are deployed for routine rabbit management activities.

#### 2.3.1. Virus

We sourced RHDV (GI.1a) from the NSW Department of Primary Industries—the equivalent vials of lyophilised RHDV that are commercially available for use by land managers (GenBank Acc# MF598301.1 [11,23]). The manufacturer states that each vial of virus has at least 30,000 ID50 units with the quantity of infectious virus titrated as part of quality control testing of this commercial product. This testing is completed following reconstitution and dilution of the lyophilised vials.

All vials were stored refrigerated prior to being reconstituted and diluted according to the manufacturer’s instructions. After reconstitution and dilution, the total volume resulting from a single vial of RHDV was 100 mL. All of the virus was applied to each of the deployment methods within 6 h of reconstitution.

#### 2.3.2. Meat Bait Group

The intention of the meat bait was to simulate the carcass of a rabbit that had died of RHDV, with its design also being simple to facilitate the potential uptake of this novel delivery method by land managers. Each meat bait consisted of 1 kg of beef liver, in a plastic container, and injected with 100 mL of reconstituted RHDV (one vial per bait). Over time, the liver leached juices into the plastic container, but it remained soaking in these juices for the duration of our experiment. This ensured that both the external and internal surfaces of the liver were contaminated with RHDV. We used beef liver as RHDV is known to remain viable and infective to rabbits in this medium for over 90 days [17]. Although we used a larger mass of liver relative to Henning et al. 2005, we note that each liver/bait was subject to an entire vial of RHDV, which contains >30,000 ID50 units.

At each time point, rabbits in the meat bait treatment group were then passively exposed with RHDV via blowflies (*Lucila cuprina*), a known RHDV vector [18,24]. A box containing 10 blowflies (sourced from South Australian Research and Development Institute Entomology) and the RHDV meat bait (250 g total per rabbit) were placed directly on top of the enclosures of the individually housed rabbits. The blowfly box was then connected to the rabbit enclosure via small diameter wire mesh that allowed the free movement of flies between the blowfly box containing the meat bait and the rabbit enclosure, but which prevented the rabbit from directly contacting or accessing the meat bait or the blowfly box it was contained in. The blowflies therefore had free access to both the liver meat bait and the rabbits for the entire duration of the experiment (i.e., for the full 14 days if the rabbit did not succumb to RHDV earlier). In this treatment group, the transmission of RHDV from the meat bait to the rabbit relied on the blowflies as mechanical vectors—equivalent to how such a meat bait would function in a field situation.

#### 2.3.3. Soil Spray Group

Similar to the meat baits, the design of the soil spray was also intended to facilitate ease-of-use by land managers. The soil spray consisted of 100 mL of reconstituted RHDV (one vial of RHDV) that was further diluted with an additional 900 mL sterile water in a hand-held spray bottle. This gave a 1000 mL total volume. We then sprayed 100 mL of this into the entrance of each experimental burrow to coat and contaminate the soil surface inside the burrows with RHDV.

At each time point, we then collected 500–900 g of soil from the surface of the inner walls of each experimental burrow into a plastic ziplock bag. Soil samples in sealed ziplock bags were then transported back to the laboratory in an airconditioned vehicle; the time between soil collection and arrival in the laboratory was <1 h. After transporting the soil back to the laboratory, we then placed 250 g in a petri dish on the floor of the enclosures of the individually housed rabbits (after removing their existing straw bedding). In this treatment group, the transmission of RHDV from the soil to the rabbit relied on the rabbits themselves becoming contaminated with the virus-laden soil, and then grooming it off through natural behaviours—again, equivalent to how such a soil spray, or similar natural RHDV transmission, would function in a field situation.

#### 2.3.4. Carrot and Oat Bait Groups

We deployed chopped carrot and whole oat baits as per the standard operating procedures for RHDV baiting [25]. We used one vial of RHDV, reconstituted and diluted as per the manufacturer’s instructions, to treat 10 kg of carrot or 5 kg of oats.

At each time point, rabbits in the carrot and oat treatment groups were exposed directly to the RHDV-treated carrots/oats by putting 400 g of treated carrot bait or 250 g of treated oat bait in a feed dish in the rabbit enclosures. In this treatment group, the transmission of RHDV from the carrots/oats relied on the rabbits directly consuming the bait, equivalent to how RHDV baiting operates for routine rabbit management activities.

#### 2.3.5. Control Group

We used one control rabbit at each time point that was exposed to all four of the deployment methods in a virus-free manner. The control rabbits’ enclosures were connected to an RHDV-free beef liver bait, with flies in an equivalent setup to the meat bait group. We additionally, placed 250 g of autoclaved soil on the floor of the rabbits’ enclosure and fed rabbits on equivalent quantities of nontreated carrots and oats.

### 2.4. Rabbit Monitoring and Trail Endpoint

We monitored all rabbits daily for signs of illness (lethargy, panting, inappetence, or death) until 14 days post exposure. Rabbits were monitored at 6 hourly intervals for the first 5 days post bait exposure and then 12 hourly thereafter until 14 days post exposure. Although rabbits exhibiting acute distress or RHDV symptoms were to be euthanised, most rabbits died between inspections without displaying observable symptoms. In such cases, we estimated the rabbit’s time to death to be the midpoint between the two inspection times (i.e., the midpoint between the last inspection time when the rabbit was found dead and the previous inspection time when it was observed alive). At 14 days post exposure, we humanely killed all surviving rabbits and performed a necropsy, paying particular attention to the liver and spleen for discolouration or enlargement, consistent with RDHV as the cause of death. Liver samples were collected for qRT-PCR to detect and quantify the amount of viral genomes to estimate viral loads.

### 2.5. Serology

We collected blood samples from each rabbit prior to their inclusion in exposure trials and then subsequently after exposure trials. All blood samples were centrifuged with sera and subsequently collected and stored at −80 °C for RHDV/RCV-A1/RHDV2 antibody testing. All serological testing for RHDV/RCV-A1/RHDV2 antibodies was conducted at the NSW Animal and Plant Health Laboratories (Elizabeth McArthur Agricultural Institute, NSW Department of Primary Industries) using three antibody ELISAs [26,27,28,29].

### 2.6. Molecular Testing

We tested all deployment methods at each time point and all rabbit liver samples collected at necropsy by qRT-PCR testing. RNA was extracted from 20–30 mg of liver tissue (rabbit or bovine) using a Maxwell 16 LEV simplyRNA tissue kit (Promega, Madison, WI, USA) as per the manufacturer’s instructions. For oat, carrot, and soil deployment methods, we added 500 µL of sterile PBS and left the bait solution to incubate at 25 °C for 5 min. A total volume of 200 µL bait solution was then used for RNA extraction using the Purelink viral RNA/DNA mini kit (Life Technologies, Carlsbad, Ottawa, ON, Canada) as per the manufacturer’s instructions. All extracts were eluted in 50 µL nuclease-free water.

Viral RNA was quantified as described previously using the SensiFAST SYBR No-Rox Kit (Bioline, Memphis, TN, USA) [30]. Full-length RHDV in vitro transcripts were used as standards for absolute quantification of viral loads. Reactions were performed in duplicate, and each run included a dilution series of the standards ranging from 1 × 10^8^–1 × 10^2^ copies/µL for quantification, a ‘no template control’ to detect contamination, and a positive control for inter-assay variation. Each reaction had a final volume of 10 µL, containing 1x SensiFAST SYBR No-Rox One-Step mix, 0.5 µM of each primer, 0.2 µL RNase inhibitor, 0.1 µL of reverse transcriptase, and 1 µL of template RNA. Cycling was performed using a CFX96 C1000 real-time PCR detection system (Bio-Rad Laboratories, Hercules, CA, USA), with reverse transcription conducted at 45 °C for 10 min, followed by denaturation at 95 °C for 5 min and then 40 cycles of 95 °C for 10 s, 63 °C for 40 sec, and 78 °C for 10 s with data acquisition. Melt curve analysis was conducted at 65–95 °C in 0.5 °C increments at 5 s per increment. Data were analysed using CFX Maestro software (Bio-Rad Laboratories, South Granville Australia) using a baseline threshold of 200.

### 2.7. Data Analysis

We conducted all data analysis in R with survival analyses completed in the *survival* package and plots prepared in *ggplot2* (R Core Team 2023) [31,32]. We estimated Kaplan–Meier survival functions for each deployment method to compare their duration (days) of infectivity to rabbits. In a similar manner, we compared the time to rabbit death (hours) between each treatment group overall and then separately for each time point. We used a log-rank test to test for differences in estimated Kaplan–Meier survival functions.

## 3. Results

### 3.1. Infectivity of Deployment Methods

**Figure 1:**
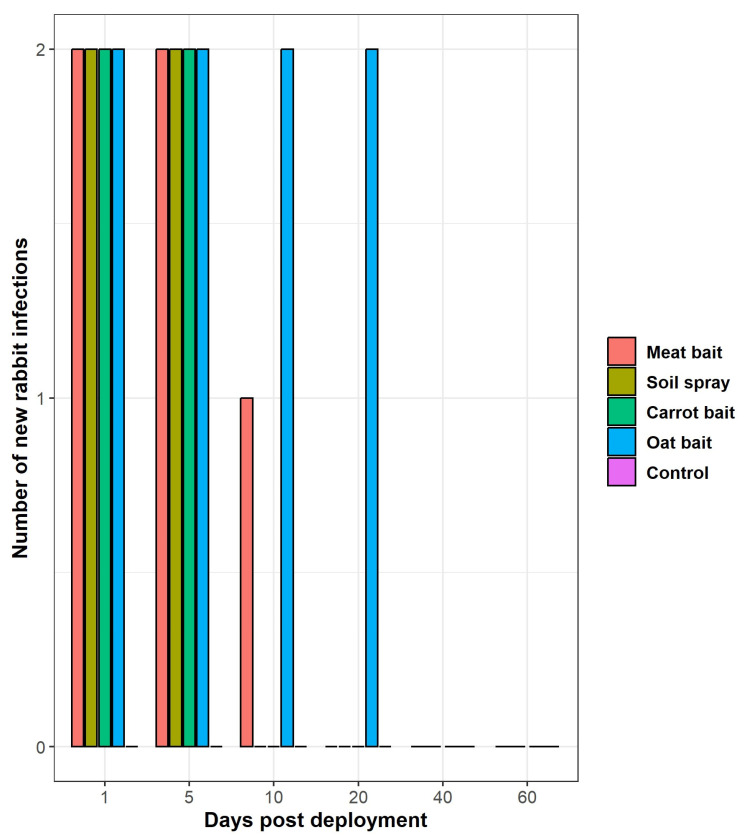
Number of new rabbit infections through time by deployment method. Adult rabbits were exposed to RHDV via one of four deployment methods (meat bait, soil spray, carrot bait, or oat bait) at six different time points post bait/spray deployment in the field (days 1, 5, 10, 20, 40, and 60 post deployment). Note that the meat bait, soil spray, and carrot bait deployment methods were all stopped at 20 days post deployment in the field; none of these three methods caused rabbit mortalities at either 10 or 20 days post deployment. For this reason, the oat bait and control were the only two deployment methods tested at days 40 and 60 post deployment in the field. Purple control bars are not evident as no rabbit mortalities occurred within the control group at any time point.

All deployment methods were terminated well before the last planned time-point trial at 120 days. Rabbits continued to become infected and succumb to RHDV-treated oat baits for up to 20 days post bait deployment (Figure 1 and Figure S1). This was twice as long as the meat bait (duration of infectivity: 10 days) and four times longer than the soil spray and carrot bait (duration of infectivity: 5 days) (Figure 1 and Figure S1).

Where the deployment method was effective, we observed 100% mortality in both rabbits, except for one rabbit (rabbit 27) exposed to the meat bait 10 days post its deployment in the field (50% mortality). All control rabbits (6/6) survived at all time-point trials (Figure S1).

At necropsy, all rabbits that died because of RHDV (20/42 rabbits total) displayed patchy livers, indicating severe disease consistent with RHDV infection (Figure S1). All rabbits showed evidence of virus, with high viral loads, in their liver via qRT-PCR.

Almost all rabbits (41/42 rabbits total) were seronegative to RHDV/RCV-A1 prior to their exposure; a single rabbit (rabbit 25) was positive to RCV-A1 prior to exposure. Twelve of 42 rabbits seroconverted during the trials, with 9 of these 12 rabbits succumbing to RHDV (Figure S1). This seroconversion in 3 surviving rabbits was only detected for RCV-A1, possibly indicating nonspecific reactivity or serological cross-reactivity.

Rabbit 27, which was exposed to meat bait at 10 days post its deployment, had detectable RHDV in its liver by qRT-PCR and seroconverted but remained alive at 14 days post exposure (Figure S1). This indicated that while exposure and infection occurred for this rabbit, no clinical disease was evident.

### 3.2. Duration of Rabbit Survival Post RHDV Exposure

**Figure 2:**
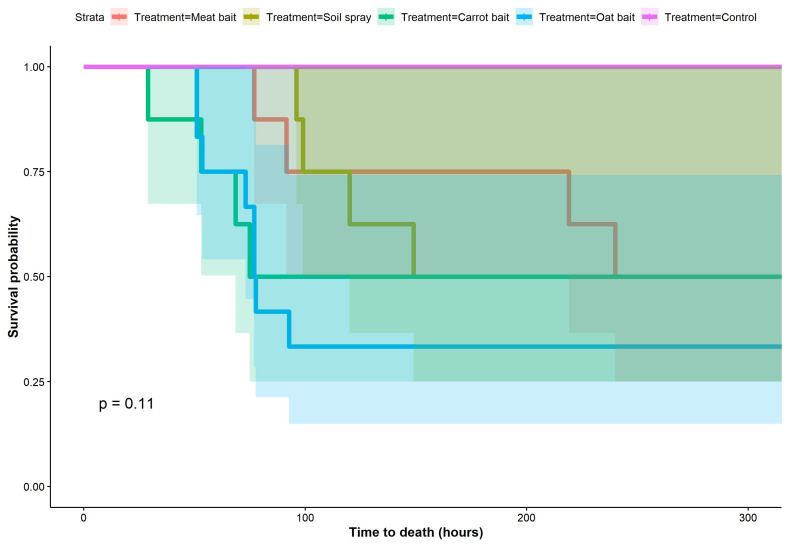
Time to rabbit death for all time points by deployment method. Adult rabbits were exposed to RHDV via one of four deployment methods (meat bait, soil spray, carrot bait, or oat bait) at six different time points post bait/spray deployment in the field (days 1, 5, 10, 20, 40, and 60 post deployment). Solid coloured lines show estimated Kaplan–Meier survival functions for each deployment method at each time point, and shaded areas show 95% confidence interval for each corresponding survival function. At each time point, two rabbits were exposed to each deployment method.

Across all time points, there was no difference in the time to rabbit death between deployment methods (Figure 2).

**Figure 3:**
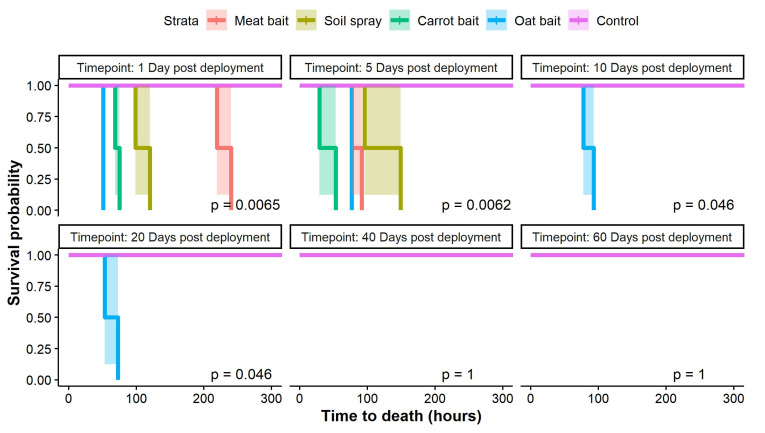
Time to rabbit death at each time point trial for each deployment method. Adult rabbits were exposed to RHDV via one of four deployment methods (meat bait, soil spray, carrot bait, or oat bait) at six different time points post bait/spray deployment in the field (days 1, 5, 10, 20, 40, and 60 post deployment). Solid coloured lines show estimated Kaplan–Meier survival functions for each deployment method at each time point, and shaded areas show 95% confidence interval for each corresponding survival function. At each time point, two rabbits were exposed to each deployment method.

However, when data were analysed separately for each time-point trial, RHDV-treated oat and carrot baits appeared to result in a shorter time to rabbit mortality (29–77 h post rabbit exposure) than the meat bait or soil spray (77–240 h post rabbit exposure) in both time-point trials where all four deployment methods killed rabbits (days 1 and 5 post bait/spray deployment; Figure 3). For the oat bait deployment method, time to rabbit mortality appeared less variable (51–93 h post rabbit exposure) across the four trials during which rabbit mortality occurred, relative to the two trials where mortality occurred within the carrot bait (29–75 h post rabbit exposure), meat bait (77–240 h post rabbit exposure), and soil spray deployment methods (96–149 h post rabbit exposure) (Figure 3; Figure S1). Most rabbits died suddenly between monitoring inspections without displaying observable symptoms. In rabbits for which symptoms were noted, we observed lethargy and inappetence. In general, earlier time points resulted in greater rabbit mortality relative to later time points across all deployment methods (Figure 3).

### 3.3. Decay of Virus in Treatments

**Figure 4:**
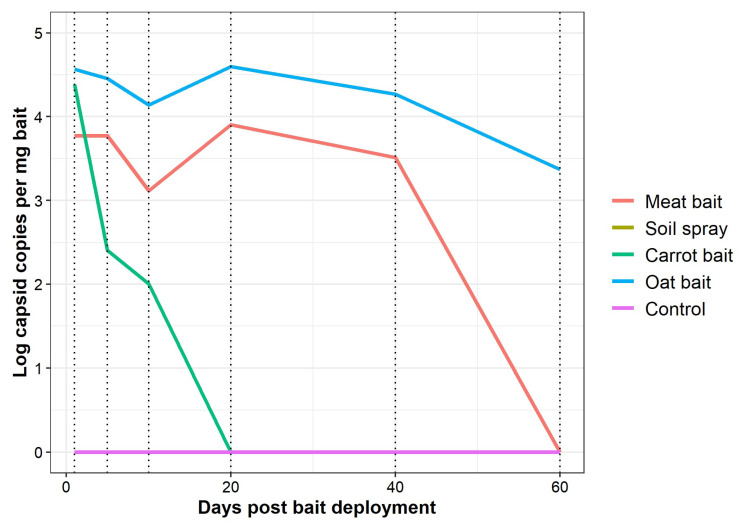
Virus copies in bait/spray deployment methods through time. RHDV-treated meat bait, soil spray, carrot bait, and oat bait were all simultaneously deployed in the environment and were subject to equivalent, natural conditions to test their respective duration of infectivity in laboratory rabbits. Meat baits, in open plastic containers, and soil spray were each deployed down 18 experimental rabbit burrows (36 burrows total). Carrot and oat baits were both deployed on the ground in open plastic containers in direct sunlight. Each deployment method was then sampled at 1, 5, 10, 20, 40, and 60 days post bait/spray deployment to test the bait/spray infectivity in rabbits. At each of these time points (represented as dotted vertical lines), a sub-sample of bait/spray was collected to quantify virus copies remaining in the bait/spray. No RHDV copies were detected in the soil spray deployment method at any time points, suggesting that qRT-PCR methods were not appropriate for this deployment method.

RHDV copies in baits decayed through time (Figure 4). All baits started with ~10^4.2395 copies per milligram of bait at 1 day post deployment. Carrot bait then showed the most rapid decay in RHDV copies through time, with no RHDV copies per milligram bait detected by 20 days post deployment. RHDV copies remained similarly high in the meat and oat bait treatments out to 40 days post deployment. However, no RHDV copies were detected in the meat bait at 60 days, suggesting that the rate of decay of RHDV in the meat bait post 40 days deployment was rapid compared to that in the oat bait, where RHDV copies remained high out to 60 days post deployment. No RHDV copies were detected in the soil spray deployment method at any time points, and no RHDV was detected in deployment methods prior to the commencement of the exposure trials.

## 4. Discussion

We tested and compared the duration of infectivity for two novel (meat bait and soil spray) and two conventional methods (carrot- and oat-treated bait) of delivering RHDV to rabbits. Our two novel methods of delivering RHDV to rabbits were intended to better replicate natural virus transmission processes and were predicted to have several benefits over the delivery of RHDV to rabbits via virus-treated carrot and oat baits, including not relying on rabbits to actively and intentionally consume the virus-treated bait, increasing the survival of the virus in the environment by reducing its direct exposure to UV radiation, and increasing the duration of time over which the virus could be transmitted to rabbits. Consistent with our predictions, both the meat bait and soil spray did prove to be an effective means of passively delivering RHDV to rabbits that did not rely on them actively or intentionally consuming a bait product. However, neither the meat bait nor the soil spray increased the survival of the virus in the environment or the duration of time over which it could be transmitted to rabbits.

All treatments effectively resulted in lethal RHDV infection, with a case fatality rate of 100% at 1 and 5 days post deployment in the field. Both meat baits and RHDV-treated oat baits were still viable at 10 days post infection, as demonstrated by mortality in the oat bait treatment and seroconversion and viral loads indicative of infection in the liver of the meat-bait-treatment rabbit. RHDV-treated oat baits were the only treatment that resulted in infection or mortality when rabbits were exposed 20 days post the deployment of baits in the field. Beyond 20 days bait deployment in the field, no RHDV infections were observed in any rabbits.

Our results are similar to previous studies investigating the duration of time over which RHDV may be transmitted to rabbits. Henning, Meers, Davies, and Morris [17] showed that bovine liver inoculated with RHDV and deployed in ambient environmental conditions can remain infective and lethal beyond 91 days deployment in the environment when rabbits are exposed via oral gavage. Similarly, under laboratory conditions, McColl et al. [33] demonstrated that following the death of a rabbit from RHDV, the virus in the carcass can remain infective and lethal for up to 20 days when rabbits are exposed via intramuscular inoculation. We followed procedures equivalent to Henning, Meers, Davies, and Morris [17] and partially similar to McColl, Morrissy, Collins, and Westbury [33]. At 40 days post deployment, our meat baits still showed high RHDV viral loads via qRT-PCR. This may suggest that if we had exposed rabbits via oral gavage or intramuscular injection, then RHDV transmission and lethal infection may have continued beyond 40 days bait deployment, similar to the aforementioned studies [17,32].

However, rabbits are strictly herbivorous and would never be naturally exposed to RHDV from an RHDV-laden carcass (after an animal had succumbed to RHD) through the oral consumption of carcass components or intramuscular inoculation of viral suspensions. Rather, natural transmission from RHDV-laden carcasses would always occur via fly or other insect vectors or direct contact with infected carcasses. When attempting to simulate such natural transmission pathways, we could not demonstrate effective RHDV transmission from inoculated bovine liver to rabbits post 10 days liver deployment in the field. This was despite qRT-PCR demonstrating that virus loads in meat baits remained high out to 40 days post bait deployment in the field. Therefore, our results may also be viewed to be contradictory to Henning, Meers, Davies, and Morris [17] and McColl, Morrissy, Collins, and Westbury [33].

Our difference in results relative to Henning, Meers, Davies, and Morris [17] and McColl, Morrissy, Collins, and Westbury [33] may have been twofold. First, the drying of the external surface of the meat bait may prevent adult flies from accessing the virus and, consequently, the effective transmission of RHDV to rabbits after only 10 days. Second, flies in our study were all male and thus did not reproduce to produce eggs or larvae (maggots). The production of larvae may be key to the transmission of RHDV from meat products. Larvae are renowned for recycling carrion, can penetrate live and dead tissue with ease, and are known to contribute to the decomposition of rabbit carcasses specifically [34]. Once fly larvae are actively feeding on and decomposing the infected tissue, RHDV transmission may then occur through three slightly different mechanisms: (1) the larvae vertically transmit RHDV to the adult fly through metamorphosis [35], and the contaminated adult fly then transmits the virus to other susceptible rabbits; (2) after metamorphosis, the adult fly emerges within the infected tissue, becomes contaminated with RHDV, and then transmits the virus to other susceptible rabbits; or (3) the decomposition of infected tissue by the larvae maintains a moist access point to the infected tissue that then facilitates access by adult flies, which become contaminated and transmit the virus to other susceptible rabbits. Irrespective of the exact mechanism through which fly transmission from infected tissue occurs, it seems likely that the ability of flies to be able to reproduce on the infected tissue, and the activity of fly larvae specifically, is of critical importance to RHDV transmission from such tissues for prolonged periods. We hypothesise that similar mechanisms are important for the transmission of RHDV from carcasses of RHDV mortalities.

Alternatively, differences in the results we observed relative to Henning, Meers, Davies, and Morris [17] and McColl, Morrissy, Collins, and Westbury [33] may be partially attributable to local environmental or climatic conditions during the period that treatments were deployed in the field. RHDV is known to be susceptible to both temperature and UV radiation, and climatic conditions are also expected to influence rabbit mortality [16,17,36]. However, local environmental conditions were mild for the duration of treatment deployment in the field, and we therefore suggest that differences in results are unlikely to be strongly driven by local environmental or climatic conditions relative to fundamental differences in transmission route and barriers to transmission (dry film/skin developing on external surface of meat bait).

The transmission of RHDV from soil to rabbits was also short lived. This was despite previous laboratory trials demonstrating that 4 weeks post the housing of infected wild rabbits in an artificial warren system maintained at 22 °C, newly introduced, susceptible rabbits experienced lethal RHDV infections, similar to our proposed route of RHDV transmission from our soil spray treatment to rabbits [37]. Similarly, Henning, Meers, Davies, and Morris [17] proposed that RHDV-impregnated cotton tape deployed in the environment simulated dried excreted virus in the field, again relying on a similar route of transmission to that proposed for our soil spray. From his RHDV-impregnated cotton tape, Henning, Meers, Davies, and Morris [17] observed lethal RHDV infection in rabbits exposed via oral gavage, after resuspension of viral particles from cotton tape, for up to 10 days post cotton deployment in the field. This was twice the duration of infectivity that we observed from our soil spray treatment, which remained infective and lethal to rabbits for only 5 days post spray deployment in the field. Irrespective of the exact duration over which such a transmission pathway may operate, our soil spray clearly has a lot of potential for the deployment of RHDV to rabbits given that all rabbits within a warren groom daily and likely enter and exit warrens with similar frequency.

Traditional methods of delivering RHDV to rabbits resulted in highly contrasting durations of transmission and rabbit mortality, with carrot baits only causing rabbit infection and mortality out to 5 days post bait deployment in the field and oat baits causing rabbit infection and mortality out to 20 days. This may be due to the capacity of oats to absorb and retain liquid, with this swelling predicted to also draw in virus particles that are then protected from desiccation as a result. In contrast, RHDV likely does not penetrate carrot baits and, rather, may only sit on the external surface of the carrot where it remains more exposed to UV radiation, desiccation, and bacterial decomposition. These results have direct implications for the use of carrot and oat baits, which are currently the major means of delivering RHDV to rabbits, and suggest that the use of oat baits may maximise the duration over which transmission can occur in the field. However, we acknowledge that carrot baits may be preferred in areas where uptake of oats by rabbits is compromised by lesser palatability and that transmission from meat baits may be prolonged if flies can complete their lifecycle on the baits. We also note that registered RHDV products currently recommend that any carrot or oat bait not consumed by rabbits 24 h after deployment be recovered and destroyed.

For all treatments where qRT-PCR was effective, viral loads remained high in baits well beyond the time point at which RHDV transmission and rabbit mortality ceased. This was expected, as infectivity depends on intact receptor-binding structures and mechanisms on the very outside of the viral capsid that decompose first, while the inner protein structures still protect the viral RNA and enable detection by qRT-PCT. Similar degradation processes and loss of infectivity were shown for RHDV even in the liver of rabbits surviving an infection [38]. Combined, these results confirm that although RHDV may remain detectable in a particular matrix for an extended period of time, this is not a reliable indicator of the persistence of infectious virus.

Based on the results of our study, we recommend similar trials be conducted in wild rabbits to confirm that meat baits and soil burrow spray can indeed initiate RHDV outbreaks in wild rabbit populations, to test for prolonged transmission time from meat baits when flies can complete their lifecycle on the bait, and to test and compare their duration of infectivity to traditional carrot and oat baits.

## 5. Conclusions

We used a series of experimental infection trials at successive time points to compare the duration of infectivity for four methods of delivering RHDV to rabbits. Meat baits and soil spray were predicted to have two key advantages over traditional carrot and oat baits: (1) they do not rely on rabbits to actively consume the bait but instead take advantage of natural RHDV transmission pathways to deliver the virus to rabbits via fly vectors and rabbit grooming behaviours, respectively; and (2) they were predicted to extend the duration of RHDV infectivity to rabbits. All bait treatments effectively killed exposed rabbits. However, soil spray and carrot baits only resulted in rabbit infection and mortality out to five days post bait deployment in the field, and meat baits only caused infection out to ten days post bait deployment. This was in contrast to oat baits, which continued to infect and kill exposed rabbits out to 20 days post bait deployment in the field. These results were in contrast to our predictions and suggest that the drying of meat baits and rabbit carcasses may create a film/skin on its external surface that then acts as a barrier to effective adult fly transmission within 10 days and that fly larvae may be critical to prolonged virus transmission from carcasses.

Based on our results, to maximise the duration of infectivity to rabbits, land managers should use RHDV-treated oat baits. However, the current approved use of RHDV-treated baits stipulates that bait remaining after 24 h should be recovered and destroyed, limiting a land managers’ ability to take advantage of the prolonged duration over which oat baits remain infective. Furthermore, the duration of infectivity of any particular RHDV delivery method may be less relevant given that the initiation of transmission and outbreaks within a warren should only require a single susceptible rabbit to consume the bait. However, a longer duration of bait infectivity may allow a greater proportion of the rabbit population to be exposed to RHDV via the bait; once a single susceptible rabbit has been exposed, the transmission of RHDV within the population should occur naturally as the infected rabbit sheds the virus and later becomes a virus-laden carcass itself. In addition, the virus load in a rabbit carcass, and possibly within a contaminated warren, vastly outnumbers what we have likely replicated here. Therefore, any additional method likely to increase the number of infections and thus fatalities in the population warrants consideration.

Our study demonstrated that meat baits and soil spray could provide additional virus delivery options to land managers that replace the need for rabbits to actively consume baits at times when they may be reluctant to do so. However, we recommend further confirmation that RHDV transmission from meat baits and soil spray is effective on wild rabbits.

## Data Availability

All raw data are provided online with the manuscript.

## Supplementary Materials

The following supporting information can be downloaded at https://www.mdpi.com/article/10.3390/v15091814/s1: Figure S1: Duration of infectivity of deployment methods. Kaplan–Meier survival curves for treatment groups. Adult rabbits were exposed to RHDV1 via one of four deployment methods (meat bait, soil spray, carrot bait, or oat bait) at six different time points post bait/spray deployment in the field (days 1, 5, 10, 20, 40, and 60 post deployment). Solid coloured lines show estimated Kaplan–Meier survival functions for each deployment method, and vertical dashed black lines show time point exposures at 1, 5, 10, 20, 40, and 60 days post bait/spray deployment. Note that curves for soil spray and carrot bait overlap perfectly, as all baits expired at the same time. At each time point, two rabbits were exposed to each deployment method.
