# Supplementary material for: Optimising the Delivery of RHDV to Rabbits for Biocontrol: An Experimental Evaluation of Two Novel Methods of Virus Delivery
Source: Viruses. 2023 Aug 25;15(9):1814. doi: 10.3390/v15091814 (PMC10536075; doi:10.3390/v15091814)
Supplement: Supplementary file 1 [file viruses-15-01814-s001.zip › viruses-2535335-supplementary.pdf]

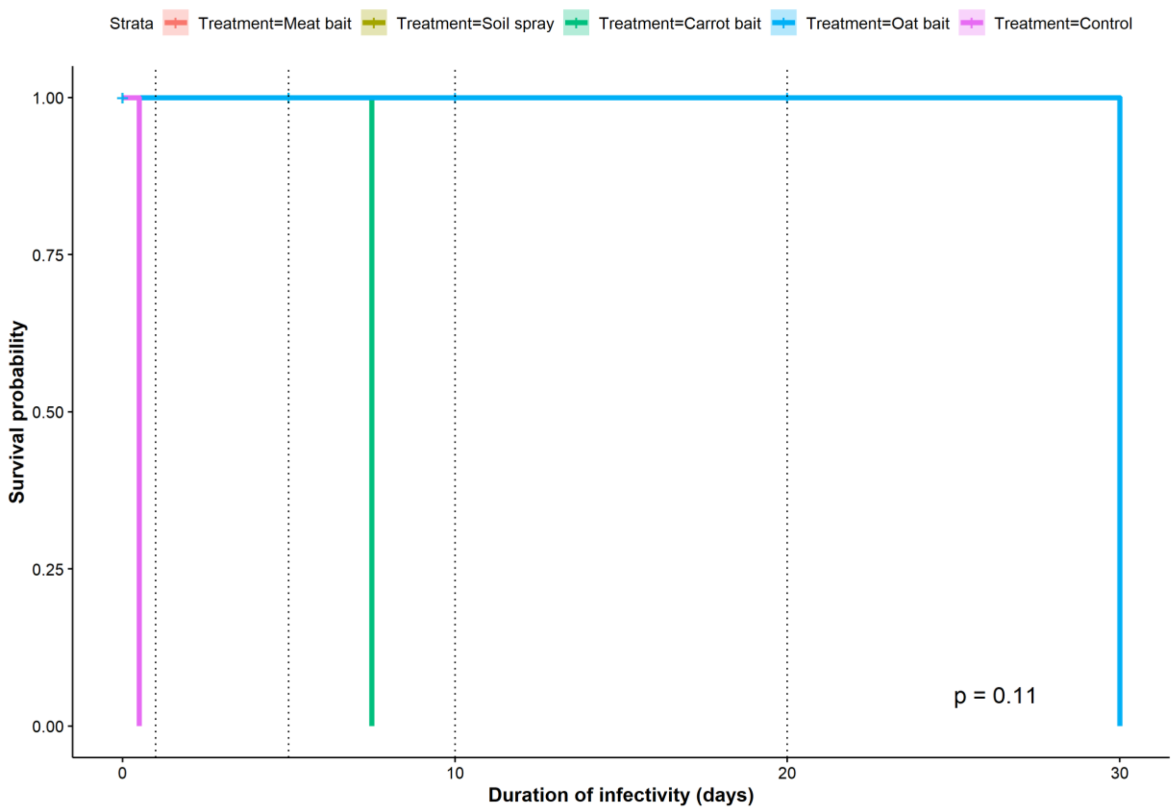


Figure S1: Duration of infectivity of deployment methods. Kaplan–Meier survival curves for treatment groups. Adult rabbits were exposed to RHDV1 via one of four deployment methods (meat bait, soil spray, carrot bait, or oat bait) at six different time points post bait/spray deployment in the field (days 1, 5, 10, 20, 40, and 60 post deployment). Solid coloured lines show estimated Kaplan–Meier survival functions for each deployment method, and vertical dashed black lines show time point exposures at 1, 5, 10, 20, 40, and 60 days post bait/spray deployment. Note that curves for soil spray and carrot bait overlap perfectly, as all baits expired at the same time. At each time point, two rabbits were exposed to each deployment method;
